# Development and Validation of Cell-Based Bioassay for the Detection of Neutralizing Antibodies to Ocrelizumab in Human Serum Using Antibody-Dependent Cell-Mediated Cytotoxicity Test in a Reporter Cell Line Expressing FcγRIIIa

**DOI:** 10.3390/antib15030046

**Published:** 2026-05-29

**Authors:** Olga Strizhakova, Grigory Poroshin, Andrei Pershin, Yana Bakhareva, Ivan Shevchenko, Ivan Lyagoskin, Rakhim Shukurov, Ravil Khamitov

**Affiliations:** JSC “GENERIUM”, 14, Vladimirskaya Street, Volginskiy 601125, Vladimir Region, Russia

**Keywords:** ADCC, neutralizing anti-drug antibodies, ocrelizumab, immunogenicity

## Abstract

Background/Objectives: Ocrelizumab is a humanized monoclonal antibody targeting CD20, approved for the treatment of adult patients with relapsing multiple sclerosis (RMS) and primary progressive multiple sclerosis (PPMS). The neutralizing activity of anti-drug antibodies (ADAs), especially neutralizing ADAs (nADAs) activity, should be examined considering that it can alter pharmacokinetic (PK) and pharmacodynamic (PD) profiles, reduce drug efficacy, and lead to life-threatening adverse events. Methods: This article presents data on the development and validation of an assay for neutralizing anti-drug antibodies (nADA) based on ADCC reporter cells for the analysis of patient sera in the context of ocrelizumab clinical studies. Results: Critical steps and conditions to minimize assay variability were identified. The lower limit of detection was 549.6 ng/mL. The cutoff for nonspecific neutralization was determined as 19.7%. The presence of 0.37–3.0 μg/mL ocrelizumab in a biological sample enables the detection of 1.1–10.0 μg/mL polyclonal anti-ocrelizumab idiotype antibodies, respectively. Conclusions: The developed method can be used for immunogenicity studies of medicinal products containing ocrelizumab.

## 1. Introduction

Ocrelizumab is a humanized monoclonal antibody of the IgG1 subclass, possessing an Fc region and designed to bind CD20, a cell surface antigen present on all B-cells except lymphoid stem cells and plasma cells. Following binding to CD20 on the surface of B-cells, the Fc portion of ocrelizumab enables it to engage Fc-mediated effector functions, which consist of specific binding of the drug to immune system cells bearing Fc receptors for IgG1 (e.g., NK cells bearing the FcγRIII receptor). This leads to activation of immune cells and eradication of ocrelizumab-bound B-cells via antibody-dependent cellular phagocytosis (ADCP), antibody-dependent cellular cytotoxicity (ADCC), complement-dependent cytotoxicity (CDC), and apoptosis. Importantly, the capacity to generate new B-cells and pre-existing humoral immunity is preserved. Furthermore, ocrelizumab does not affect innate immunity or total T-cell counts.

Assessment of immunogenicity is a crucial and mandatory component of the safety evaluation of biopharmaceutical drugs [1,2]. The immune system’s response to an administered drug is influenced by numerous factors, including the patient’s genetic background, the pathogenesis of the underlying disease, concomitant conditions, dosing regimen, route and method of administration, the drug’s properties and characteristics, and concomitant use of other medications [3]. A key step in this evaluation is assessing the neutralizing activity of anti-drug antibodies (ADAs), as neutralizing ADAs (nADAs) can alter pharmacokinetic (PK) and pharmacodynamic (PD) profiles, reduce drug efficacy, and lead to life-threatening adverse events [4,5,6].

There are two main types of assays for detecting neutralizing antibodies: ligand-binding assays and cell-based assays. Ligand-binding assays typically assess the binding of the drug to its target in vitro; cell-based assays utilize cells expressing the drug target, with drug activity evaluated based on changes in cellular function. Cellular responses may more closely reflect the mechanisms by which nADAs affect the drug in vivo [6,7]. Moreover, the current trend in selecting a neutralizing antibody assay format favors using the therapeutic mechanism of action as the primary selection criterion [1,8,9]. Therefore, to detect nADAs against ocrelizumab in human serum samples, a cell-based test evaluating ADCC was selected. The method utilized a reporter cell line expressing FcγRIIIa (V158 variant)—a polymorphic form of the Fcγ receptor crucial for ADCC, where the Valine (V) allele at position 158 (V158) is one of the most common and active variants influencing antibody binding. WIL2-S B-lymphocyte cells expressing the CD20 cell surface antigen were used as target cells.

Ocrelizumab, by binding to CD20 on the surface of target cells, activates the FcγIIIa receptor signaling pathway in effector cells, leading to luciferase production. ADCC activity correlates with the activation of the NFAT transcription factor. The luminescence signal is proportional to ADCC activity and inversely proportional to the level of nADAs present in serum samples.

## 2. Materials and Methods

### 2.1. Cell Cultures and Reagents

Cell Cultures: Human B-lymphocyte cell line, WIL2-S (ATCC CRL-8885, Manassas, VA, USA) (target cells); ADCC Bioassay Effector cells (Promega^®^, Fitchburg, WI, USA, G7017) (effector cells). Pooled inactivated normal human serum (Merck Millipore, Spruce, ST, USA No. S1-100 ML), Fetal bovine serum (FBS, heat-inactivated), HyClone, Logan, UT, USA (No. FBS-HI-11A), Bio-Glo™ Luciferase Assay System, Promega^®^ (No. G7940) or homemade substrate for luciferase with D-luciferin and ATP. Liquid culture medium RPMI1640 without phenol red, (Capricorn Scientific GmbH, Ebsdorfergrund, Germany, RPMI-XRXA). Rat polyclonal antibodies to ocrelizumab, GENERIUM JSC, GNR-085/Rat pAb/050924. Polyclonal antibodies to tocilizumab, GENERIUM JSC, AB087010819. Ocrevus^®^ (Roche, Basel, Switzerland) and ocrelizumab biosimilar (GENERIUM JSC).

Individual serum samples were obtained from healthy donors who provided voluntary informed consent for sample collection and inclusion of their analysis results in the study.

### 2.2. Preparation of Model Samples

Positive model samples (MS) with high, medium, and low concentrations of polyclonal antibodies (PC)—“high positive sample” (HPS), “medium positive sample” (MPS), and “low positive sample” (LPS)—as well as a negative model sample (NS), were prepared using pooled heat-inactivated serum from healthy donors. Rat polyclonal antibodies to ocrelizumab were added to the positive model samples at concentrations of 30 μg/mL (HPS); 10 μg/mL (MPS_10); 5 μg/mL (MPS_5); 3 μg/mL (LPS_3); and 1 μg/mL (LPS_1). No antibodies were added to the negative model sample.

### 2.3. Screening Test

All samples were analyzed in triplicate. Ocrelizumab at a concentration of 10 ng/mL was added to the test and model serum samples and incubated for (60 ± 5) minutes at (37 ± 1) °C in an atmosphere of (5 ± 0.5)% CO_2_ to allow formation of nADA–ocrelizumab complexes. After incubation, test and model samples were transferred to a white 96-well culture plate (No. 236105 Nunc™ Thermo Fisher Scientific, Waltham, MA, USA), and target and effector cells were added according to the plate layout. Incubation was carried out for (24 ± 1) hours at (37 ± 1) °C and 5% CO_2_.

Luminescence levels in the wells were measured using a plate spectrophotometer with an integration time of 1000 ms. The analytical signal is proportional to ADCC activity and inversely proportional to nADA levels. The mean chemiluminescent value, standard deviation (SD), and coefficient of variation (%CV) were calculated for each test sample and quality control. The coefficient of variation should not exceed 20%. If the coefficient of variation was ≤20%, the percentage neutralization for the sample was calculated using:

$$\%N=100\times \left(1-\frac{{\bar{A}}_{BS}\;-\;{\bar{A}}_{NCK}}{{\bar{A}}_{NC}\;-\;{\bar{A}}_{NCK}}\right),$$

where ${\bar{A}}_{NC}$—mean signal of the negative model sample (NMS) at the minimum required dilution (MRD);

${\bar{A}}_{BS}$—mean signal of the individual biological sample at the MRD;

${\bar{A}}_{NCK}$—mean signal of the NMS at the MRD for wells without added drug.

### 2.4. Determination of Neutralizing ADA Titer

Titration was performed while maintaining a constant proportion of biological matrix in the test medium. For titer determination, the following formula was used:

$$Titer=R_{H}+\left(\frac{{(N}_{H}-{CP}_{\%N})\times (R_{L}-R_{H})}{\left(N_{H}-N_{L}\right)}\right),$$

where $R_{H}$—maximum dilution factor of the positive model sample, where the mean percentage neutralization exceeds the nonspecific neutralization cutoff;

${CP}_{\%N}$—nonspecific neutralization cutoff;

$R_{L}$—minimum dilution factor of the positive model sample where the mean percentage neutralization is below the nonspecific neutralization cutoff;

$N_{H}$—percentage neutralization value for the positive model sample at the maximum dilution that exceeds the nonspecific neutralization cutoff;

$N_{L}$—percentage neutralization value for the positive model sample at the minimum dilution that is below the nonspecific neutralization cutoff.

### 2.5. Determination of Nonspecific Neutralization Cutoff

The nonspecific neutralization value separating positive and negative test samples (nonspecific neutralization cutoff) was evaluated using a panel of serum samples from 50 healthy volunteers. Each serum sample was analyzed at the minimum required dilution across 6 independent analytical runs by two analysts on different days. In each analytical run, the mean luminescence value for replicates was calculated, the coefficient of variation for each test sample was determined, and the percentage neutralization was calculated. The resulting percentage neutralization values for the study panel were analyzed for normality using the D’Agostino and Pearson normality tests. The cutoff was set at a level allowing up to 5% false positive results [10], ensuring detection of true positive samples with low antibody concentrations. After normality was assessed, outliers (values significantly deviating from the rest) were identified using the interquartile range. Following outlier exclusion and confirmation of normal distribution, the nonspecific neutralization cutoff was calculated using:

$${CP}_{\%N}=100-10^{\bar{log\%N}-2.33\times {SD}_{log\%N}},$$

where $\bar{log\%N}$—mean of the decimal logarithm of the %N for the panel of individual biological samples at the MRD;

2.33—0.99 quantile of the standard normal distribution;

${SD}_{log\%N}$—standard deviation of the log%N for the panel of individual biological samples at the MRD.

### 2.6. Specificity

The ability of the method to unequivocally assess the analyte in the presence of other components that may interfere with the reaction. To evaluate the specificity of the nADA detection and titer determination methods, model samples free of ADAs to ocrelizumab but containing nonspecific ADAs were analyzed in triplicate at the MRD. For each sample, the mean luminescence value (^−^LU), coefficient of variation (%CV), and %N were calculated. A %N value in model samples with nonspecific antibodies was expected to be less than or equal to the nonspecific neutralization cutoff (${CP}_{\%N}$).

### 2.7. Precision

A measure of how closely independent results from repeated measurements align. Precision was assessed both within a single analytical cycle (AC) (repeatability) and across ACs (reproducibility).

### 2.8. Repeatability of the Screening Test

Assessed based on the analysis of 7 independent measurements of three positive (HPS, MPS, and LPS) model samples (MS) at the MRD within a single AC. The coefficient of variation (%CV) was calculated based on the neutralization percentage values in the MS. A CV level not exceeding 20% was considered acceptable.

### 2.9. Repeatability of the Titer Determination

Repeatability was assessed based on three independent replicate titer measurements in three positive MS at the MRD at a single AC. The median titer values were calculated across all independent replicates from the three experiments. A difference between the maximum and minimum titers of each MS of no more than 2.5 times the calculated median was considered acceptable.

### 2.10. Reproducibility of the Screening Test

Two analysts evaluated the method in 12 independent experiments, assessing three positive MS at the MRD. The %CV for each PMO was determined based on the calculated values of the neutralization percentage across the experiments. A CV not exceeding 25% was considered acceptable.

### 2.11. Reproducibility of the Titer Determination

Reproducibility was assessed based on 6 independent titrations of positive MS (HPS, MPS, and LPS) performed at different AC by two analysts. A difference between the maximum and minimum titers of each MS of no more than 2.5 times the calculated median was considered acceptable.

### 2.12. Lower Limit of Detection

The lower limit of detection (LLOD) was calculated based on 6 independent experiments across 3 analytical runs using:

$${\;LLOD}_{method}\;=\bar{{LLOD}_{test}}+3\times SD,$$

where $\bar{{LLOD}_{test}}$*—*average LLOD value from 6 independent tests, ng/mL;

SD*—*standard deviation.

The LLOD for each experiment was determined as:

$${\;LLOD}_{test}\;=\left(\;\frac{C}{Titer}\times MRD\right),$$

where C—concentration of neutralizing ADAs in the positive MS, ng/mL;

Titer*—*calculated titer of the positive MS for that test;

MRD—minimum required dilution.

### 2.13. Matrix Effect (Selectivity)

The matrix effect was investigated using biological serum samples from 10 healthy donors, spiked with ADAs to a concentration near the LLOD. The acceptability criterion was achieving a %N above the nonspecific neutralization cutoff (CP_%N_) in at least 80% of the tested model samples.

### 2.14. Hook Effect

Very high concentrations of the analyte can lead to false negative results. The potential for a hook effect was investigated using samples spiked with ADAs concentrations eight times higher than that in the high-positive samples. The acceptability criterion was achieving a %N exceeding the nonspecific neutralization cutoff (CP_%N_) in spiked samples.

### 2.15. Drug Tolerance

Drug tolerance was evaluated using pooled healthy donor serum samples containing 10, 3.3, 1.1, 0.37, 0.12, 0.04, 0.013, and 0 μg/mL of polyclonal anti-ocrelizumab antibodies. These were spiked with pooled healthy donor serum containing 12, 6.3, 1.5, 0.75, 0.37, and 0 μg/mL of ocrelizumab. Samples were incubated at room temperature for (60 ± 5) minutes to allow antibody binding, then frozen at (−80 ± 10) °C overnight to simulate sample processing. After analysis, the mean luminescence value (^−^LU) and the %N were calculated for each sample.

### 2.16. Sample Stability During Storage and Analysis

To confirm stability during storage, transport, and analysis, samples were investigated under various storage conditions:

- Freezing at ≤−70 °C, thawing at room temperature, incubation for 20 h in a refrigerator at (5 ± 3) °C;
- Freezing at ≤−70 °C, thawing at room temperature, refreezing for 20 h at ≤−20 °C;
- Freezing at ≤−70 °C, thawing at room temperature (20 ± 5) °C, and refreezing at ≤−70 °C (2 cycles);
- Freezing at ≤−70 °C, thawing at room temperature (20 ± 5) °C, and refreezing at ≤−70 °C (3 cycles).

For each model sample, the recovery rate (%R) relative to freshly prepared samples were calculated:

$$\%R=\frac{\bar{{\%N}_{after}}}{\bar{{\%N}_{before}}}\times 100,$$

where $\bar{{\%N}_{after}}$*—*mean %N after processing;

$\bar{{\%N}_{before}}$*—*mean %N for fresh samples.

The acceptability criterion was a recovery rate %R within the range of 80–125%.

### 2.17. Statistical Analysis

Experimental data were processed using GraphPad Prism 9 (GraphPad Software Inc., La Jolla, CA, USA) and Microsoft Office Excel 2016. For each test sample, the mean optical density (OD) value for replicates was calculated, and the CV was determined.

## 3. Results

During method development, factors influencing assay sensitivity and specificity were evaluated to optimize ADCC activity.

**Figure 1:**
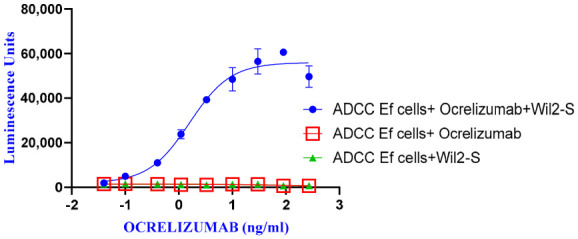
Ocrelizumab ADCC activity assessment during ADCC complex formation studies.

To confirm the necessity of ocrelizumab binding to target cells for effector cell activation (ADCC Ef cells), we incubated ocrelizumab only with effector cells (in the absence of target cells). The data demonstrated that effector cell activation requires ocrelizumab binding to target cells. As expected, ocrelizumab did not activate ADCC Bioassay Effector cells in the absence of target cells (WIL2-S), and target cells without added ocrelizumab did not activate ADCC (Figure 1).

**Figure 2:**
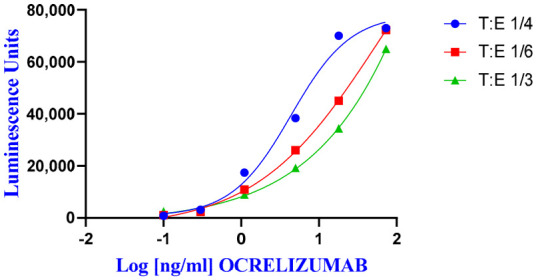
Ocrelizumab ADCC activity assessment during target-to-effector cell ratio studies.

To optimize ADCC activity, various target-to-effector cell ratios were evaluated: 1:3, 1:4, and 1:6. For each cell ratio, the drug response factor, or signal-to-noise ratio (ratio of the signal at the highest drug concentration to the signal at the lowest drug concentration), was calculated. A dose-dependent increase in luminescence signal was observed (Figure 2). The maximum signal-to-noise ratio of 26.0 was observed at a target-to-effector cell ratio of 1:4 (6.0 × 10^3^ target cells/well and 2.5 × 10^4^ effector cells/well).

**Figure 3:**
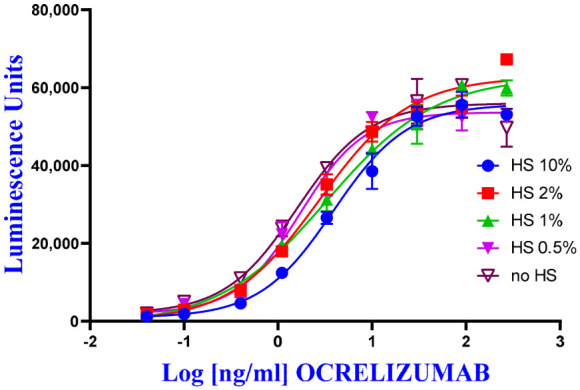
Ocrelizumab ADCC activity assessment during human serum (HS) nonspecific interference studies.

To assess the nonspecific interference of human serum on ocrelizumab ADCC activity, studies were conducted with the addition of 10%, 2%, 1%, 0.5%, and 0% pooled human serum (ocrelizumab concentration range 270 ng/mL to 0.04 ng/mL). A minimal effect of serum on ocrelizumab ADCC activity was established (Figure 3). To balance assay response and sensitivity, the addition of 10% pooled human serum (equivalent to a 10-fold sample dilution) was selected.

**Figure 4:**
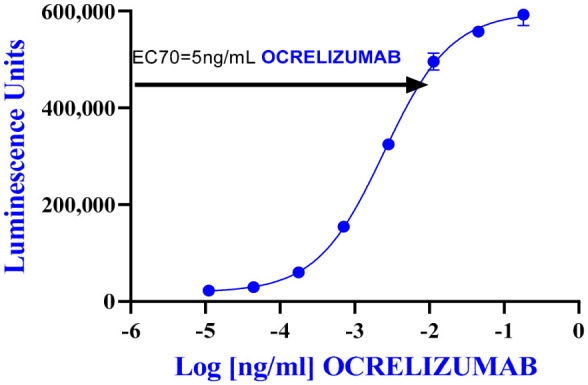
Optimal ocrelizumab level determination.

To determine the optimal ocrelizumab level, experiments were conducted using the established parameters: a target-to-effector cell ratio of 1:4 and an assay medium containing 10% human serum. A maximum drug response factor of 26 (ratio of mean luminescence in wells with ocrelizumab to mean luminescence in wells without drug) was achieved at approximately 100 ng/mL, followed by a plateau. Ocrelizumab at 5 ng/mL induced a 20-fold induction of response compared to background signal, equivalent to approximately 70% of the maximal effective concentration (EC70) (Figure 4).

Since clinical samples containing neutralizing ADA to ocrelizumab were not available during development and validation, we used rat polyclonal antibodies to ocrelizumab (GENERIUM JSC, Moscow, Russia).

### 3.1. Determination of Nonspecific Neutralization Cutoff

**Figure 5:**
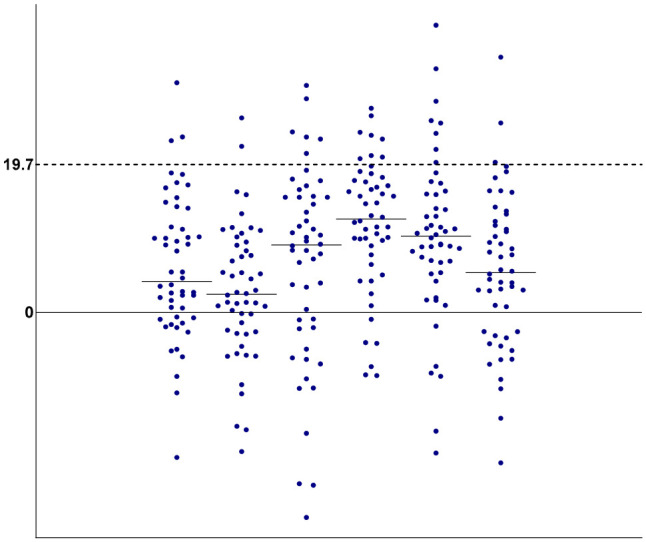
Visualization of the nonspecific neutralization cutoff.

Based on the analysis of samples from 50 healthy volunteers across independent analytical runs, the nonspecific neutralization cutoff was determined to be 19.7% (Figure 5).

Each point represents an individual healthy donor serum sample (%N). Dashed line indicates the cutoff level. Solid-line indicates zero neutralization level.

### 3.2. Determination of LLOD and Acceptable Titer Range for LPS and HPS

The LLOD for neutralizing ADAs was determined by analyzing positive model samples with 1 μg/mL of ADA. It was estimated as 549.6 ng/mL (Table 1).

### 3.3. Specificity

Ocrelizumab is a humanized IgG1 monoclonal antibody. Thus, to confirm specificity, we investigated nonspecific anti-tocilizumab antibodies, as tocilizumab is also a humanized IgG1 monoclonal antibody. Negative results were obtained for all model samples containing nonspecific ADAs (Table 2).

### 3.4. Precision

Precision test was based on the mean $\bar{\%N}$ and CV for %N determination in model samples (Table 3).

Precision for titer determination was assessed based on the deviation of the maximum and minimum titers for each positive model sample from the calculated median value (Table 4).

### 3.5. Matrix Effect (Selectivity)

Analysis of sera spiked with 1.0 μg/mL anti-ocrelizumab antibodies (LPS level) in screening and confirmatory assays alongside samples without added antibodies showed that in 10 out of 10 cases, the %N for patient serum samples without added nADAs did not exceed the nonspecific neutralization cutoff (CP_%N_) and in 10 out of 10 cases, the %N for patient serum samples with added nADAs was greater than (CP_%N_).

### 3.6. Hook Effect Evaluation

Model samples containing neutralizing ADAs at concentrations exceeding those in HPS were tested. The %N value for all samples exceeded the nonspecific neutralization cutoff (CP_%N_) (Table 5). Thus, no hook effect was observed.

### 3.7. Drug Tolerance

Free drug present in the biological sample is important in assay setup as it significantly influences sensitivity. For drug tolerance determination, the calculated %N for each dilution of the positive MS was compared to the nonspecific neutralization cutoff (CP_%N_). The presence of 0.75 μg/mL ocrelizumab in a sample allows detection of 3.3 μg/mL of polyclonal anti-ocrelizumab antibodies. The presence of 0.37–3.0 μg/mL ocrelizumab in a sample enables detection of 1.1–10.0 μg/mL of polyclonal anti-ocrelizumab antibodies, respectively (Table 6).

### 3.8. Robustness to Replacement of Key Components

To test the robustness, the luciferase detection reagent was replaced and the originator drug was used instead of the GENERIUM JSC ocrelizumab. Positive MS tested with replaced reagents (each one separately) remained positive and the maximum %CV did not exceed 20%.

### 3.9. Sample Stability During Storage and Analysis

The recovery rate R values during model sample stability assessment ranged from 93 to 121% (Table 7), meeting the established acceptability criteria and thus confirming the stability of samples under the specified storage conditions.

## 4. Discussion

A cell-based ADCC assay for detecting neutralizing anti-drug antibodies was developed and validated, based upon one of the mechanisms of action of ocrelizumab.

The use of rat polyclonal antibodies as a surrogate for human ADA does not completely reflect the complexity of human immunogenic responses. Therefore, the assay will undergo further validation using human clinical samples.

Standardization and validation of ADCC assays are fundamental, as any change in methodology can impact assay sensitivity and specificity [11]; therefore, we aimed to standardize all stages.

The selection of validation parameters was based upon the characteristics of the chosen analytical methods and the drug product. During development, we identified critically important steps: (1) formation of the ADCC complex; (2) target-to-effector cell ratio; (3) detection of the NFAT-dependent response upon FcγRIIIa receptor activation; and (4) potential nonspecific interference of human serum with ocrelizumab ADCC activity and formation of nADA–ocrelizumab complexes.

In this work, we experimentally confirmed that effector cell activation requires ocrelizumab binding to target cells.

Currently, three types of effector cells are commonly used in bioassays for ADCC evaluation: primary natural killer cells from donors (primary NK cells with FcγRIIIA+), modified NK-92 cells expressing FcγRIIIA, and modified Jurkat T-cells expressing FcγRIIIA/NFAT-RE/luc2. Modified effector cells were developed to enhance the accuracy and reliability of classical ADCC bioassays using NK cells [12]. In our study, we used modified Jurkat T-cells with FcγRIIIA/NFAT-RE/luc2, optimizing the target-to-effector cell ratio.

Given that the neutralizing ADA assay will be used for testing serum samples, we paid particular attention to evaluating the nonspecific effect of human serum on ADCC activity.

Biological components present in serum can non-specifically modulate the cellular response. To reduce matrix effects, sample dilution in assay medium is applied; however, excessive sample dilution can reduce sensitivity. We found that a 10-fold sample dilution (10% serum concentration) does not affect ocrelizumab ADCC activity and allows sensitivity to be maintained. Thus, we established the minimum required dilution.

It is well-known that therapeutic proteins present in serum can influence assay sensitivity. Drug interference can potentially lead to false-negative results in nADA detection. Evaluating assay sensitivity in the presence of expected levels of interfering therapeutic protein, also known as drug tolerance, is crucial for understanding the suitability of the method for nADA detection [13]. Analysis of spiked samples demonstrated limitations of our method: it allows detection of 1.1 μg/mL of polyclonal anti-ocrelizumab idiotype antibodies in the presence of 0.37 μg/mL of ocrelizumab. The sensitivity was 549.6 ng/mL. Similar data were obtained by other researchers [14] using MEC-2 cells (a chronic B-cell leukemia cell line) pre-labeled with calcein AM as target cells, and natural killer (NK) cells modified for stable expression of the Fc-gamma IIIa_F158 receptor as effector cells.

The core principle of the method is the detection of the unbound (free) ADA in blood serum. It is well-known that therapeutic proteins present in serum can limit assay sensitivity, potentially leading to false-negative results in nADA detection. The serum concentration of ADA is closely associated with the concentration of the free drug. The detectable serum ADA level increases when the concentration of the free drug declines or when the ADA concentration exceeds the drug level. Therefore, it is possible to miss the detection of nADA at early onset. Furthermore, the presence of ADAs could not be linked to infusion-related reactions [13].

Evaluating assay sensitivity in the presence of expected levels of interfering therapeutic protein, also known as drug tolerance, is crucial for understanding the suitability of the method for nADA detection [14]. Analysis of spiked samples demonstrated limitations of our method: it allows detection of 1.1 μg/mL of polyclonal anti-ocrelizumab idiotype antibodies in the presence of 0.37 μg/mL of ocrelizumab. The sensitivity was 549.6 ng/mL. Similar data were obtained by other researchers [15] using MEC-2 cells (a chronic B-cell leukemia cell line) pre-labeled with calcein AM as target cells, and natural killer (NK) cells modified for stable expression of the Fc-gamma IIIa_F158 receptor as effector cells.

These results indicate that this method can be used for immunogenicity studies of medicinal products containing ocrelizumab.

## 5. Conclusions

A cell-based method for detection of neutralizing antibodies against ocrelizumab via antibody-dependent cell-mediated cytotoxicity using a reporter cell line expressing FcγRIIIa was developed and successfully validated in accordance with the guidelines of the Russian Ministry of Health, FDA, and EMEA.

During validation testing, the following were determined: the nonspecific neutralization cutoff (19.7%), the lower limit of detection (549.6 ng/mL), and drug tolerance. The precision was confirmed for both the screening test and titer determination steps. The absence of matrix and hook effects was demonstrated, as well as robustness, specificity, and sample stability. Through validation, we confirmed the suitability of the method for testing clinical samples. Additional characterization of the method should be conducted utilizing positive human serum test samples.

## Figures and Tables

**Table 1:** Lower limit of detection determination for neutralizing ADAs.

NO.	Titer	${LLOD}_{test}$, ng/mL	SD	${LLOD}_{method}$, ng/mL
1.	59.2	169.0	105.1	549.6
2.	76.6	130.6		
3.	27.2	367.5		
4.	74.8	367.5		
5.	54.2	184.6		
6.	53.7	186.3		

**Table 2:** Specificity testing.

${CP}_{\%N}$ = 19.7	MS with Anti-Tocilizumab ADA in Human Serum			MS with Anti-Ocrelizumab ADA in Human Serum		
	30 μg/mL	10 μg/mL	30 μg/mL	30 μg/mL	10 μg/mL	3 μg/mL
$\bar{\%N}$	5	5	5	107	105	104
%CV	3	3	7	3	6	4
%N ≥ ${\;CP}_{\%N}$?	No	No	No	Yes	Yes	Yes

**Table 3:** Precision evaluation for the screening test.

Value	Model Samples		
	HPS	MPS	LPS
Repeatability			
$\bar{\%N}$	101.7	100.2	99.0
%CV	0.9%	1.0%	1.2%
Reproducibility			
$\bar{\%N}$	103.0	101.6	100.1
%CV	4%	3%	3%

**Table 4:** Precision evaluation for the titer determination.

Value	Model Samples		
	HPS	MPS	LPS
Repeatability			
Median	1797.6	811.4	201.2
Reference interval for titer measurement	8722-1395	3782-605	400-64
Max titer	4530.2	924.7	270.6
Min titer	1770.6	700.1	150.8
Meets the criteria?	Yes	Yes	Yes
Reproducibility			
Median	3488.7	1512.8	159.9
Reference interval for titer measurement	8722-1395	3782-605	400-64
Max titer	5513.5	2080.7	219.6
Min titer	1441.3	896.7	103.5
Meets the criteria?	Yes	Yes	Yes

**Table 5:** Hook effect evaluation.

	LU n1	LU n2	LU n3	$\bar{LU}$	%CV	${CP}_{\%N}$	
Negative control	151,443	150,480	160,090	154,004.3	3%	19.7	
ADA Concentrarion, μg/mL	LU n1	LU n2	LU n3	$\bar{LU}$	%CV	%N	%N > ${CP}_{\%N}$?
60	33,115	30,683	33,033	32,277.0	4%	106	Yes
120	32,766	31,052	32,393	32,070.3	3%	106	Yes
240	31,663	25,965	30,908	29,512.0	10%	109	Yes

**Table 6:** Assessment of the effect of drug present in the biological sample.

Ocrelizumab (μg/mL)	Anti-Ocrelizumab Polyclonal Antibodies (μg/mL)							
	10	3.3	1.1	0.37	0.12	0.04	0.013	0
12	−14.3	−17.4	−13.5	−11.0	−11.0	−9.6	−8.1	0
6	−9.8	−4.7	−0.8	−3.8	2.5	−6.5	−2.8	0
3	70.8	−7.0	−0.4	−4.7	−0.05	0.3	3.1	0
1.5	80.4	3.9	−6.7	−4.4	−6.3	2.6	3.31	0
0.75	79.2	71.6	1.6	−0.2	−3.0	−5.5	−6.7	0
0.37	77.8	76.8	55.5	8.9	4.5	0.8	−10.2	0
0	−8.2	−16.5	−19.5	−14.7	−13.4	−2.5	3.3	0

**Table 7:** Sample stability assessment under different storage conditions.

Treatment	Variable	Samples				
		HPS_30	MPS_10	MPS_5	LPS_3	LPS_1
Fresh made	$\bar{\%N}$	101.0	100.4	97.9	101.6	81.0
	%R	-	-	-	-	-
Freezing at ≤−70 °C (storage period: 50 days)	$\bar{\%N}$	100.0	100.1	103.3	101.6	79.4
	%R	99	100	105	100	98
Freezing at ≤−70 °C, thawing, 20 h at (5 ± 3) °C	$\bar{\%N}$	100.5	102.4	104.4	100.2	97.4
	%R	99	102	107	99	120
Freezing at ≤−70 °C, thawing, refreezing for 20 h at (≤20) °C	$\bar{\%N}$	101.1	101.9	104.0	94.5	97.7
	%R	100	102	106	93	121
Freezing at ≤−70 °C, thawing, refreezing at (≤70) °C, thrice.	$\bar{\%N}$	98.9	102.5	103.1	95.1	92.7
	%R	98	102	105	94	114

## Data Availability

The original contributions presented in this study are included in the article. Further inquiries can be directed to the corresponding author.

## Abbreviations

The following abbreviations are used in this manuscript:

%N	Percent neutralization
%R	Percent recovery
AC	Analytical cycle
ADA	Anti-drug antibodies
ADCC	Antibody-dependent cellular cytotoxicity
ADCP	Antibody-dependent cellular phagocytosis
CDC	Complement-dependent cytotoxicity
CV	Coefficient of variation
CP_%N_	Nonspecific neutralization cutoff
HPS	High positive sample
LLOD	Lower limit of detection
LPS	Low positive sample
LU	Luminescence Unit
MS	Model samples
MPS	Medium positive sample
MRD	Minimum required dilution
nADA	Neutralizing anti-drug antibodies
PC	Positive control
PD	Pharmacodynamic
PK	Pharmacokinetic
SD	Standard deviation
